# Factors associated with occupation changes after pregnancy/delivery: result from Japan Environment & Children’s pilot study

**DOI:** 10.1186/s12905-018-0575-3

**Published:** 2018-06-05

**Authors:** Reiko Suga, Mayumi Tsuji, Rie Tanaka, Eiji Shibata, Masayuki Tanaka, Ayako Senju, Shunsuke Araki, Seiichi Morokuma, Masafumi Sanefuji, Masako Oda, Nathan Mise, Yosuke Baba, Mina Hayama-Terada, Koichi Kusuhara, Hiroshi Mitsubuchi, Takahiko Katoh, Toshihiro Kawamoto

**Affiliations:** 1 Regional Centre for Japan Environment & Children’s Pilot Study, University of Occupational and Environmental Health, Japan, 1-1, Iseigaoka, Yahata-nishi-ku, Kitakyushu-shi, Fukuoka, 807-8555 Japan; 2 Department of Environmental Health, School of Medicine, University of Occupational and Environmental Health, Japan, 1-1, Iseigaoka, Yahata-nishi-ku, Kitakyushu-shi, Fukuoka, 807-8555 Japan; 3 Department of Obstetrics and Gynecology, School of Medicine, University of Occupational and Environmental Health, Japan, 1-1, Iseigaoka, Yahata-nishi-ku, Kitakyushu-shi, Fukuoka, 807-8555 Japan; 4 Fukuoka Institute of Occupational Health, 1-11-27, Nanokawa, Minami-ku, Fukuoka-shi, Fukuoka, 815-0081 Japan; 5 Department of Pediatrics, School of Medicine, University of Occupational and Environmental Health, Japan, 1-1, Iseigaoka, Yahata-nishi-ku, Kitakyushu-shi, Fukuoka, 807-8555 Japan; 6 Regional Centre for Japan Environment & Children’s Pilot Study, Kyushu University, 3-1-1, Maidashi, Higashi-ku, Fukuoka-shi, Fukuoka, 812-8582 Japan; 7 Regional Centre for Japan Environment & Children’s Pilot Study, Kumamoto University, 1-1-1, Honjo, Chuo-ku, Kumamoto-shi, Kumamoto, 860-8556 Japan; 8 Department of Environmental and Preventive Medicine, School of Medicine, Jichi Medical University, 3311-1, Yakushiji, Shimotsuke-shi, Tochigi, 329-0498 Japan; 9 Department of Obstetrics and Gynecology, School of Medicine, Jichi Medical University, 3311-1, Yakushiji, Shimotsuke-shi, Tochigi, 329-0498 Japan; 10 Regional Centre for Japan Environment & Children’s Pilot Study, Jichi Medical University, 3311-1, Yakushiji, Shimotsuke-shi, Tochigi, 329-0498 Japan; 11 Osaka Centre for Cancer and Cardiovascular Disease Prevention, 1-3-2, Nakamichi, Higashinari-ku, Osaka-shi, Osaka, 537-0025 Japan

**Keywords:** Occupation changes, After pregnancy/delivery, Employment, Mother

## Abstract

**Background:**

In Japan, although the number of females who continue to work after marriage has recently increased, the proportion of those working while parenting their infants is still not clearly increasing, indicating that it is still difficult for them to continue working after delivery. The present study aimed to clarify factors influencing females’ continuation of work, using data obtained by continuously following up the same subjects and focusing on occupation changes, family environments, and the type of employment after pregnancy or delivery.

**Methods:**

Based on the results of the questionnaire survey, which was conducted involving 164 participants at 4 universities, as part of the Japan Environment and Children’s Pilot Study (JECS Pilot Study) led by the Ministry of Environment and the National Institute for Environmental Studies, the occupational status was compared between the detection of pregnancy (weeks 0 to 7) and 1 year after delivery.

**Results:**

<Non-regular employees> compared with <regular employees> changed their occupations significantly more frequently (OR = 5.07, 95% CI = 2.57–10.01, *P* < 0.001). Furthermore, on examining <non-regular employees> in detail, occupation changes were particularly marked among <part-time and short-term contract employees> (OR = 12.48, 95% CI = 4.43–35.15, *P* < 0.001). This tendency was especially shown among <<those engaged in specialized or technical work> > (OR = 10.36, 95% CI = 1.59–67.38, *P* = 0.014) and < <those engaged in clerical work or management> > (OR = 15.15, 95% CI = 2.55–90.17, *P* = 0.003).

**Conclusions:**

Analysis revealed that the type of employment, rather than the category of occupation, was associated with the continuation of work after pregnancy or delivery more closely, as <non-regular employees> compared with <regular employees> continued to work less frequently. Furthermore, on comparison of the category of occupation among <regular employees>, <<those engaged in specialized or technical work> > and < <those engaged in clerical work or management> > were shown to be more likely to continue to be engaged in the same occupation after pregnancy or delivery. These differences may be related to availability of the child-care leave program and other support resources, therefore, it may be important to establish social systems that enable all females, to use these support resources if they wish, and actively work, while delivering and parenting their children.

## Background

With the promotion of their social participation, a large number of females are engaged in occupations today, and the number of those who continue to work after marriage is increasing in Japan. In the Status of Working Women 2016 [1], the size of the female labor force was estimated at 28,830,000, and females accounted for 43.4% of the total working-age population in 2016. However, various problems closely related to women’s psychological, physical health, or social situation may interrupt the continuity of work or career path. Especially, marriage and childbirth are significant life events for women. In the traditional Japanese culture, women work until marriage, and afterward, they are expected to take on the responsibility of maintaining the home, usually at the expense of their work outside the home. An awareness survey of the Japanese woman’s career in the area 2015 [2] revealed that 44.2% of those surveyed held the traditional idea that the wife should maintain the house. In addition, 62.0% of the respondents confirmed that the wife should be in the house when the children are young. The Fifteenth Japanese National Fertility Survey, 2015 [3] found that almost all women who continued to work after having children received help from their mothers (children’s grandmothers) or they used some kind of institution or facility for child care. In other words, the lack of these supports may affects the continuation of work more frequently. Women may have inadequate access to daycare or nursery options, and they may be criticized by surrounding people, such as grandparents and co-workers who disagree with their choice to work, leading them to experience both psychological stress and physical stress. Depression following pregnancy and childbirth is also a common component of the stress experienced by young mothers. However, the rising costs of day-to-day expenses and education for a growing family also exert economic pressure. A survey on the re-employment of women after child rearing in 2008 [4] found that the most common and direct reason for women to resume work after child rearing was “economic necessity,” which was reported by 39.7% of the participants. Similarly, in the 2015 survey [3], 52.1% of the women responded with the same answer. Evidently, women wanting to quit their job may not be able to do so easily.

In this regard, the female labor force participation rate showed an M-shaped curve, with 2 peaks in their twenties and fifties and a depression in their thirties. This tendency is particularly marked in Japan compared with other countries. According to an age-based classification in the White Paper on Gender Equality 2015 [5], such a depression in the female labor force participation rate in the thirties is the second most marked in Japan on a global comparison, following South Korea. Although the degree of the depression is decreasing yearly, the White Paper on the National Lifestyle 2006 [6] explains this by increased numbers of unmarried individuals and couples without a child/children, suggesting that increases in the proportion of females working while parenting their infants have been limited. The necessity of promoting females’ participation in the labor force in consideration of their deliveries is particularly high in Japan compared with other countries, and this issue has been examined in a number of studies. Higuchi et al. [7] compared the female employment rate among Japan, the United States, and Great Britain, and reported that the rate among those without a spouse was the highest in Japan, nevertheless the rate after delivery was the lowest, indicating that the influences of marriage and delivery preventing them from continuing to work are the most marked. Takeishi [8] noted that, despite various approaches to support females to continue to work while parenting their children since the enforcement of the Equal Employment Opportunity Law, their career patterns have not markedly changed, as the proportion of those who continue to work after delivery has remained at 15%. Furthermore, Imada [9] compared multiple cohorts varying in age, and reported that a large number of females resigned from work even in young cohorts. More recently, the Fifteenth Japanese National Fertility Survey, 2015 [3] found that the rate of continuing work after the birth of the first child was 38.3% in 2010–2014, and this increased by ~ 10% in 2005–2009. However, this survey also found that 46.9% of the women who worked before childbirth retired from work after the birth of their first child, and this group made up 33.9% of the total female population (http://www.ipss.go.jp/ps-doukou/j/doukou15/NFS15_report4.pdf, p.52, Fig. II-4-6) [3].

Based on the Vital Statistics 2014 [10], the total fertility rate, indicating the number of children delivered by a single female throughout her life, is limited to 1.42. In Japan’s aging society with a declining fertility rate, increasing both the female labor force participation and fertility rates is an urgent issue; however, these findings indicate that females still face difficulty in continuing to work after delivery. In order to address this, it may be important to clarify and resolve factors associated with such difficulty. Although various factors are likely to be associated, the majority of previous studies were short-term using specific existing panel data or those obtained through Internet-based interviews, for instance, a comprehensive study on challenges in supporting females to work while parenting [11]. Furthermore, the number of those in which data were directly and continuously collected from the same subjects, focusing on multiple factors, has been limited. Considering such a situation, the present study aimed to clarify factors influencing females’ continuation of work, using data obtained by continuously following up the same subjects and focusing on occupation changes, family environments, and the type of employment after pregnancy or delivery.

## Methods

### Japan environment and Children’s pilot study (JECS pilot study) design

The Japan Environment and Children’s Pilot Study (JECS Pilot Study) is an epidemiological birth cohort study conducted by the Ministry of Environment and the National Institute for Environmental Studies, at 4 universities (Jichi Medical University, the University of Occupational and Environmental Health Japan, Kyushu University, and Kumamoto University), since 2008. Pregnant women were recruited from cooperating gynecological clinics between February 2009 and March 2010.The way of the agreement was received by a document after informed consent. All participants were residents of the target area (Honshu 1 area and Kyushu 3 areas where the university is located). The pilot study included 453 women and their children, and they were followed until their children were 13 years old.

Biological samples (e.g., blood, urine, and hair) were collected in cooperating recruited gynecology clinics twice during pregnancy and at the regular check-up for their children in the first month. Questionnaire surveys were administered at the first, second, and third trimesters, and every six months following the birth of the child, beginning with the first month. The questionnaire is written in Japanese and is self-administered. They were mailed to the subjects’ houses, and completed questionnaires were returned by mail. A single detailed survey examined child development, home environment, etc. The JECS aimed to examine the effects of environmental factors on children’s health and development using a combination of surveys and biological samples. The JECS Pilot Study confirms the feasibility of the content and method of the JECS [12].

### The Study’s sample

**Fig. 1:**
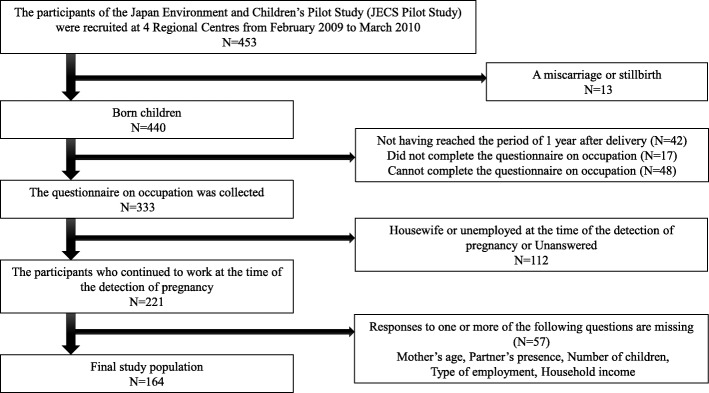
The process selecting participants

The study subjects were a part of the JECS pilot study, and the final sample for the present study was 164 (Fig. 1). The participants were adult females in their twenties to forties, who continued to work after pregnancy.

Mother’s age, partner’s presence, number of children, mother’s education, and household income were obtained using the questionnaires at the first trimester and the second to third trimester of pregnancy. These questionnaires included questions about basic information such as age and family composition, lifestyle factors such as conditions during pregnancy, and environment such as residential status; the utilized data were extracted from these. The occupation-related data was obtained from the questionnaire at 1.5 years after delivery. This questionnaire included an occupational status section. Two times of mother’s occupational statuses, the detection of pregnancy (weeks 0 to 7) and 1 year after delivery, were asked in the occupational status section, because women generally returned to work after their 1-year child-care leave [13]. The occupational status section included the following information: occupational classification code, type of employment, number of working days per week, presence or absence of shifts or night shift, and presence of maternity leave or child-care leave. The rate of returned completed the questionnaires at the first trimester and second to third trimester of pregnancy was 76.8%, and the rate for the completed occupational status section was 88.7%.

### Analysis method

Their occupations were classified using the major categories defined in the Japanese Standard Classification of Occupations 2009 [14], and the occupational classification codes were selected and filled out by the participants. These codes were categorized from 1 to 6, according to the relevant occupations category based on the Japanese Standard Classification of Industries [15]. Furthermore, employees satisfying the following 3 points were classified as <regular employees>: “there is no fixed period of labor contract,” “the prescribed working time is full time,” “direct employment.” All others were classified as <non-regular employees>. These classifications were made based on the guidelines of The Vision of the Preferred Way of Working 2012 [16].

The category of occupation was compared between the detection of pregnancy (weeks 0 to 7) and 1 year after delivery to classify the participants who continued to be engaged in the same occupation as an “unchanged group” and those who resigned from work or changed their occupations as a “changed group”. We categorized the mother’s age as “20’s”, “30’s”, or “40’s” and number of children as “0”, “1”, “2”, or “3 or over”, and used them as categorical variables. Educational level was assessed using the following categories: junior high school, high school, colleges of technology, professional training colleges, junior college, university, and graduate school. Household income was assessed using the following categories: < 2 million yen; 2.0–3.9 million yen, 4.0–5.9 million yen, 6.0–7.9 million yen; 8.0–9.9 million yen; 10.0–11.9 million yen; 12.0–14.9 million yen, 15.0–19.9 million yen, and ≥ 20 million yen. For the analysis, the 164 participants with all the basic information and occupation data were set as the population.

The relationship between an occupation change 1 year after delivery and the type of employment was examined by performing multivariate logistic regression analysis, adjusting for the mother’s age and number of children. All analyses were performed using STATA Version 14 (Stata Corporation, College Station, TX, USA), and all *p*-values presented are two-sided (α = 0.05).

## Results

Table 1 shows basic information regarding the participants. The absence of a spouse/partner was rare among them, and the proportion of those without a spouse was limited to 1.8%. The number of children excluding the child born during this study was 0 in 48.2% and 1 or more in 51.8%. Regarding education, the rate of high school, professional training colleges, and University were large, at 20% or more. For household income, the category of 2.0–3.9 million yen had the highest proportion (34.1%), followed by 4.0–5.9 million yen (26.2%) (Table 1).

**Table 1:** Characteristics of the study participants

	All participants		Those engaged in specialized or technical work		Those engaged in clerical work or management		Those engaged in sales		Those engaged in service industry, security, forwarding, cleaning, packaging, or other business		Those engaged in manufacturing, agriculture, forestry, or fisheries		Those engaged in unclassifiable businesses or students	
	(*N* = 164)		(*N* = 43)		(*N* = 55)		(*N* = 17)		(*N* = 35)		(*N* = 7)		(*N* = 7)	
	N	%	N	%	N	%	N	%	N	%	N	%	N	%
Mother’s age*			43		55		17		35		7		7	
20’s	50	30.5	8	18.6	12	21.8	10	58.8	16	45.7	2	28.6	2	28.6
30’s	108	65.9	33	76.7	40	72.7	7	41.2	19	54.3	5	71.4	4	57.1
40’s	6	3.7	2	4.7	3	5.5	0	0.0	0	0.0	0	0.0	1	14.3
Partner’s presence)^a^														
Yes	161	98.2	43	100.0	55	100.0	17	100.0	32	91.4	7	100.0	7	100.0
No	3	1.8	0	0.0	0	0.0	0	0.0	3	8.6	0	0.0	0	0.0
Number of children**)^b^														
0	79	48.2	24	55.8	20	36.4	7	41.2	20	57.1	5	71.4	3	42.9
1	53	32.3	13	30.2	23	41.8	6	35.3	9	25.7	0	0.0	2	28.6
2	22	13.4	5	11.6	8	14.5	3	17.6	5	14.3	0	0.0	1	14.3
3 or over	10	6.1	1	2.3	4	7.3	1	5.9	1	2.9	2	28.6	1	14.3
Mother’s education														
Junior high school	4	2.4	0	0.0	1	1.8	1	5.9	2	5.7	0	0.0	0	0.0
High school	42	25.6	1	2.3	12	21.8	9	52.9	14	40.0	3	42.9	3	42.9
Colleges of Technology	0	0.0	0	0.0	0	0.0	0	0.0	0	0.0	0	0.0	0	0.0
Professional training colleges	45	27.4	21	48.8	9	16.4	3	17.6	10	28.6	1	14.3	1	14.3
Junior college	29	17.7	6	14.0	13	23.6	2	11.8	5	14.3	1	14.3	2	28.6
University	40	24.4	12	27.9	19	34.5	2	11.8	4	11.4	2	28.6	1	14.3
Graduate school	4	2.4	3	7.0	1	1.8	0	0.0	0	0.0	0	0.0	0	0.0
Household income														
< 2 million yen	5	3.0	0	0.0	0	0.0	1	5.9	3	8.6	1	14.3	0	0.0
2.0–3.9 million yen	56	34.1	10	23.3	15	27.3	9	52.9	15	42.9	3	42.9	4	57.1
4.0–5.9 million yen	43	26.2	11	25.6	14	25.5	4	23.5	11	31.4	1	14.3	2	28.6
6.0–7.9 million yen	28	17.1	11	25.6	10	18.2	2	11.8	4	11.4	1	14.3	0	0.0
8.0–9.9 million yen	20	12.2	8	18.6	9	16.4	1	5.9	1	2.9	1	14.3	0	0.0
10.0–11.9 million yen	8	4.9	2	4.7	4	7.3	0	0.0	1	2.9	0	0.0	1	14.3
12.0–14.9 million yen	2	1.2	0	0.0	2	3.6	0	0.0	0	0.0	0	0.0	0	0.0
15.0–19.9 million yen	2	1.2	1	2.3	1	1.8	0	0.0	0	0.0	0	0.0	0	0.0
≥ 20 million yen	0	0.0	0	0.0	0	0.0	0	0.0	0	0.0	0	0.0	0	0.0

*We categorized the mother’s age as “20’s”, “30’s”, “40’s”, and used it as a categorical variable

**We categorized the number of children as “0”, “1”, “2”, or “3 or over”, and used it as a categorical variable

^a^A common-law marriage is included in the partner’s presence

^b^Any child born during this study was not included in the number of children

The participants were classified based on the category of occupation before pregnancy for comparison between the detection of pregnancy (weeks 0 to 7) and 1 year after delivery to examine their tendencies in relation to the continuation of work.

When focusing on factors possibly influencing the continuation of work, the presence of a spouse/partner or number of children was not associated with occupation changes. On the other hand, the participants with a child/children, compared with those without them, changed their occupations significantly less frequently. This tendency was more marked among those with a larger number of children. On comparison based on the type of employment, <non-regular employees> compared with <regular employees> changed their occupations significantly more frequently (OR = 5.07, 95% CI = 2.57–10.01, *P* < 0.001) (Table 2). There was no significant difference between mother’s education and household income.

**Table 2:** The relation between the continuation of work and each factor (*N* = 164)

	Comparison between 0 and 7 weeks of pregnancy and 1 year after childbearing					
	All	No change	Change^a^	OR^b^	95% CI	*P*-value
Mother’s age						
20’s	50	22	28	1.00		
30’s	108	56	52	0.73	0.37–1.43	0.359
40’s	6	3	3	0.79	0.14–4.28	0.78
Partner’s presence						
Yes	161	79	82	1.00		
No	3	2	1	0.39	0.03–4.48	0.447
Number of children						
0	79	28	51	1.00		
1	53	29	24	0.46	0.22–0.95	0.035
2	22	16	6	0.21	0.07–0.61	0.004
3 or over	10	8	2	0.14	0.03–0.72	0.019
Type of employment						
Regular employee	91	60	31	1.00		
Non-regular employee	73	21	52	5.07	2.57–10.01	< 0.001
Mother’s education						
Junior high school	4	2	2	1.00		
High school	42	18	24	1.27	0.16–9.95	0.821
Colleges of Technology	0	0	0			
Professional training colleges	45	27	18	0.65	0.08–5.10	0.686
Junior college	29	16	13	0.86	0.11–7.05	0.891
University	40	15	25	1.74	0.22–13.79	0.599
Graduate school	4	3	1	0.43	0.02–9.05	0.59
Household income						
< 2 million yen	5	2	3	1.00		
2.0–3.9 million yen	56	19	37	1.33	0.20–8.65	0.768
4.0–5.9 million yen	43	25	18	0.51	0.08–3.37	0.481
6.0–7.9 million yen	28	17	11	0.46	0.07–3.26	0.438
8.0–9.9 million yen	20	12	8	0.48	0.06–3.65	0.481
10–11.9 million yen,	8	4	4	0.73	0.07–7.21	0.791
12–14.9 million yen	2	1	1	0.78	0.03–21.94	0.884
15–19.9 million yen	2	1	1	0.81	0.03–23.16	0.9
≥ 20 million yen	0	0	0			

^a^Retired or changed occupation

^b^The mother’s age was always included in the models as covariates.

Tables 3 and 4 present the findings of the analyses conducted by adjusting for mothers’ age and number of children, which exhibited significant differences in the findings presented in Table 2. Table 3 shows that the relationship between change in occupation and type of employment. Furthermore, on examining <non-regular employees> in detail, occupation changes were particularly marked among <part-time and short-term contract employees> (OR = 12.48, 95% CI = 4.43–35.15, *P* < 0.001). A similar tendency was also observed among a non-significant number of <temporarily-dispatched employees>. In contrast, <those who are self-employed> showed a tendency to continue to work without changing their occupations (Table 3).

**Table 3:** The relation between a change in occupation and the type of employment

	Comparison 0-7 weeks of pregnancy and 1 year after childbearing					
	All	No change	Change^a^	OR^b^	95% CI	*P*-value
Regular employee	91	60	31	1.00		
Non-regular employee						
Temporarily dispatched employees	12	1	11	25.04	2.90–215.93	0.003
Part-time or short-term contract employees	44	9	35	12.48	4.43–35.15	< 0.001
Those who are self-employed	16	11	5	1.61	0.45–5.80	0.463
Others (contract employees)	1	0	1	–	–	–

^a^Retired or changed occupation

^b^The mother’s age and number of children were always included in the models as covariates

**Table 4:** The relation between a change in occupation and the type of employment classified by occupation

	Major categories defined in the Japanese Standard Classification of Occupations	Comparison 0-7 weeks of pregnancy and 1 year after childbearing					
		All	No change	Change^a^	OR^b^	95% CI	*P*-value
Those engaged in specialized or technical work	B	43	29	14			
Regular employee		35	27	8	1.00		
Non-regular employee		8	2	6	10.36	1.59–67.38	0.014
Those engaged in clerical work or management	A, C	55	26	29			
Regular employee		34	21	13	1.00		
Non-regular employee		21	5	16	15.15	2.55–90.17	0.003
Those engaged in sales	D	17	6	11			
Regular employee		5	2	3	1.00		
Non-regular employee		12	4	8	2.54	0.10–62.32	0.567
Those engaged in service, security industry, forwarding, cleaning, packaging, or other business	E, F, K	35	14	21			
Regular employee		15	9	6	1.00		
Non-regular employee		20	5	15	9.87	1.24–78.38	0.030
Those engaged in manufacturing, agriculture, forestry, or fisheries	G, H	7	3	4			
Regular employee		2	1	1	1.00		
Non-regular employee		5	2	3	1.80	0.02–141.05	0.791

^a^Retired or changed occupation

^b^The mother’s age and number of children were always included in the models as covariates

Table 4 shows to clarify whether or not the category of occupation influences such differences in the continuation of work between <regular employees> and < non-regular employees>, the 2 types of employment were compared based on the category of occupation. The above-mentioned tendency, <non-regular employees> compared with <regular employees> changed their occupations, was particularly marked among <<those engaged in specialized or technical work> > (OR = 10.36, 95% CI = 1.59–67.38, *P* = 0.014) and < <those engaged in clerical work or management> > (OR = 15.15, 95% CI = 2.55–90.17, *P* = 0.003). Among <regular employees> engaged in these categories, the proportion of those who continued to work compared with those who did not was higher. In contrast, the proportion of <non-regular employees> who continued to work was low, revealing more marked differences between the 2 types of employment in these categories compared with others (Table 4).

While <regular employees> compared with <non-regular employees> were shown to continue to be engaged in the same occupation more frequently after pregnancy, the influence of the category of occupation on the tendency of <regular employees> to continue to work remained unclear. Therefore, <<those engaged in specialized or technical work> > and the other category of occupation were compared, revealing that occupation changes were the most frequent among <<those engaged in sales> > (OR = 10.34, 95% CI = 1.07–100.35, *P* = 0.044), followed by <<those engaged in clerical work or management> > (OR = 2.89, 95% CI = 0.92–9.08, *P* = 0.069) (data not shown).

In contrast, <non-regular employees> did not show significant differences among different categories of occupation; in all categories, the number of those who changed their occupations compared with those who continued to be engaged in the same occupation after pregnancy weeks 0 to 7 was higher.

## Discussion

To clarify factors preventing a large number of females from continuing to work and leading them to resign or change their occupations after pregnancy or delivery as a major life event, the present study focused on the categories of occupation before and after delivery. On comparison between <regular employees> and < non-regular employees>, the proportion of those who continued to work was lower among the latter. Among <regular employees>, <<those engaged in specialized or technical work> > and < <those engaged in clerical work or management> > were shown to be more likely to continue to be engaged in the same occupation after pregnancy. The present study is significant in that, unlike previous studies, the present study collected the data directly and was not based on secondary data. The use of data directly collected from subjects facilitated the clarification of associated factors while relating the category of occupation to the type of employment, and the study may have significance in this respect.

In a previous study conducted in 2008 [11], focusing on the influences of the type of employment, the proportion of females who resigned from work during their first pregnancies was 29.6% among <regular employees>, while the value was markedly higher among <non-regular employees>, at 48.3%. Regarding differences in the likelihood of continuing to work among different categories of occupation, Takeishi (2009) et al. reported that <<those engaged in specialized or technical work> > accounted for 43.5% of all those who continued to work, followed by <<those engaged in clerical work>> [8].

The tendency of <regular employees> to continue to work may be associated with more stable work environments for them compared with <non-regular employees>, including the term of employment and salaries, in addition to the availability of the child-care leave program as a major system to support females to work while parenting. Limited-term contract workers, such as <non-regular employees>, had previously been excluded from the scope of the child-care leave program until the Revised Child Care and Family Care Leave Law was enacted in April 2005 to enable them to use this system if they meet certain requirements. However, as shown in a research report (2012) [17], the rate of child-care leave program use by <non-regular employees> remained at 11.5, and 60.0% of them resigned from work despite law enactment in 2005, while these values of <regular employees> were 60.8 and 32.4%, respectively, suggesting a close association of the availability of the child-care leave program with the difference in the continuation of work between them.

On the other hand, it should be noted that the program is not available for all <regular employees>. According to the White Paper on the National Lifestyle (2006) [6], more than 40% of the <regular employees> who continued to work without using the program mentioned “workplace atmospheres” or “work situations” as reasons for avoiding using it. This indicates that a large number of females working as <regular employees> experience psychological and physical stress due to the difficulty in obtaining co-workers’ understanding regarding availing the child-care leave program, and therefore, they are forced to resign or change their occupation.

Other factors influencing the continuation of work may include: the presence of a spouse/partner and number of children. Regarding the former, Higuchi et al. [7] reported that the proportion of employed wives was higher in multi- compared with 2-generation family households. In other words, support from the family may reduce the physical stress experienced by women, and they may continue to work. However, in the present study, significant differences were not observed at this point, as the absence of a spouse/partner was rare among the participants, indicating the necessity of analysis involving an increased number of subjects. Furthermore, regarding the number of children, the tendency to continue to work was more marked among those with a larger number of children. Sato et al. [18] similarly reported that both <regular employees> and < limited-term contract employees> with a child/children, compared with those without them, continued to work more frequently, and the proportion of such individuals was as high as 45.6% among those with 2 or more children. This confirms that the presence of a large number of children is not necessarily a negative factor for females to work. In addition, other factors related to the individual are related to the reason for occupation changes. A survey on the employment structure [19] in Japan revealed that both high education and income affected the continuity of women’s work. However, this tendency was not observed in the present study.

To explain the high likelihood of <<those engaged in specialized or technical work> > and < <those engaged in clerical work or management> > continuing the same category of occupation after pregnancy or delivery, occupation selection trends among females after graduation from university should be examined. According to the Status of Working Women 2013 [20], the proportion of <<those engaged in specialized or technical work> > after graduation was the highest, at 36.6%, followed by <<those engaged in clerical work>>, at 31.9%. Based on this, females with higher occupational awareness levels may be more likely to select these categories of occupation to advance their career, and maintain them even after delivery to meet their wishes.

Shigeno et al. [21] noted that the actual functioning of support systems influences females’ behaviors related to their first deliveries, as the probability of workers, excluding <<those engaged in independent business>>, delivering the first child is reduced by half or more if the child-care leave program is unavailable. In short, not only to encourage females to continue to work, but also to increase the fertility rate, the establishment of systems that enable all females, regardless of whether regular or non-regular employees, to use the child-care leave program if they wish by stabilizing working conditions for <non-regular employees> and promoting an understanding of the necessity of using such programs in the workplace may be an important challenge.

In a study by Imada et al. [22], the effect on the continuation of work was the most favorable when 3 support resources were simultaneously used: the child-care leave program, nursery school services, and support from relatives. To resolve the actual situation in which it is only possible for females working as <regular employees> engaged in limited categories of occupation to continue to work, combined support approaches are indispensable. Through these approaches, it may be important to establish social systems that enable females to select lifestyles they desire, and actively work while delivering and parenting their children.

### Limitations

There are several limitations in the present study. First, this study used a comparatively small sample size, which leads to broad confidence intervals. Therefore, there is a possibility that it does not provide precise estimates. It is necessary to analyze a larger number of participants and to improve the reliability of data. In the future, we plan to investigate the same through the nationwide “The Japan Environment and Children’s Study.” Second, the present analyses did not include the mothers’ individual factors in detail, such as psychological, physical, and social factors. Specifically, we did not pay attention to the individual diseases related to physical stress, and therefore, we could not confirm their influence. We also plan to consider this issue in the nationwide survey. Third, the possibility of a selection bias might not be negated. This pilot study did not use an established study method, and it involved many complicated investigations. Therefore, the participants included in the pilot study may have been more cooperative and highly conscious. Furthermore, as the study employed a self-report questionnaire, the way people respond to questions may have differed across participants. In addition, this study includes several participants whose responses to one or more of the questions are missing. Participants may not have responded because they did not wish to offer negative responses. There is a possibility of a selection bias by excluding participants who did not respond intentionally. Fourth, since a self-reported questionnaire was used, it is impossible to totally escape the possibility of social desirability bias. However, because the JECS Pilot Study included a face-to-face survey and the use of official documents, it is assumed that the presence of social desirability bias was unlikely.

## Conclusions

Based on these results, the type of employment such as <regular employees> and < non-regular employees>, rather than the category of occupation, may be associated with the continuation of work after pregnancy or delivery more closely. The existing literature suggested a close association of the availability of the child-care leave program with the difference in the continuation of work between them. Therefore, it may be important to establish social systems that enable all females, regardless of the type of employees, to use the child-care leave program and other support resources if they wish, and actively work they desire, while delivering and parenting their children.
